# Special vulnerability of somatic niche cells to transposable element activation in *Drosophila* larval ovaries

**DOI:** 10.1038/s41598-020-57901-2

**Published:** 2020-01-23

**Authors:** Olesya A. Sokolova, Elena A. Mikhaleva, Sergey L. Kharitonov, Yuri A. Abramov, Vladimir A. Gvozdev, Mikhail S. Klenov

**Affiliations:** 1 Department of Molecular Genetics of the Cell, Institute of Molecular Genetics, Russian Academy of Sciences, 2 Kurchatov Sq., 123182 Moscow, Russian Federation; 2 Present Address: Laboratory of Postgenomic Research, Engelhardt Institute of Molecular Biology, Russian Academy of Sciences, 32 Vavilova St., 119991 Moscow, Russian Federation

**Keywords:** Cell biology, DNA damage and repair, Piwi RNAs, Stem-cell differentiation

## Abstract

In the *Drosophila* ovary, somatic escort cells (ECs) form a niche that promotes differentiation of germline stem cell (GSC) progeny. The piRNA (Piwi-interacting RNA) pathway, which represses transposable elements (TEs), is required in ECs to prevent the accumulation of undifferentiated germ cells (germline tumor phenotype). The soma-specific piRNA cluster *flamenco* (*flam*) produces a substantial part of somatic piRNAs. Here, we characterized the biological effects of somatic TE activation on germ cell differentiation in *flam* mutants. We revealed that the choice between normal and tumorous phenotypes of *flam* mutant ovaries depends on the number of persisting ECs, which is determined at the larval stage. Accordingly, we found much more frequent DNA breaks in somatic cells of *flam* larval ovaries than in adult ECs. The absence of Chk2 or ATM checkpoint kinases dramatically enhanced oogenesis defects of *flam* mutants, in contrast to the germline TE-induced defects that are known to be mostly suppressed by *сhk2* mutation. These results demonstrate a crucial role of checkpoint kinases in protecting niche cells against deleterious TE activation and suggest substantial differences between DNA damage responses in ovarian somatic and germ cells.

## Introduction

Many fundamental questions concerning the mechanisms of self-renewal and differentiation of stem cells are addressed using *Drosophila* oogenesis as a model^1^. *Drosophila* ovaries consist of ovarioles, chains of egg chambers connected to the germarium, which houses germline stem cells (GSCs). A microenvironment of somatic cells known as a niche regulates GSC state via different cell signaling pathways^1–3^. The ovarian niche includes terminal filament (TF) cells, cap cells (CCs), and escort cells (ECs). GSCs directly contact CCs and the most anterior ECs, which prevent GSC differentiation by secreting decapentaplegic (Dpp) and glass bottom boat (Gbb) protein ligands^4–7^. These ligands interact with GSC surface receptors and activate BMP signaling, which represses transcription of the *bam* gene required for GSC differentiation. After GSC division, one of the daughter cells retains its stem state, whereas the other one leaves the self-renewal niche and begins to differentiate into a cystoblast, which then divides and differentiates to form a cyst of germ cells surrounded by somatic follicle cells. A special marker of GSCs and cystoblasts is the spectrosome, a cytoplasmic body, which transforms into a branching structure called the fusome connecting the dividing germ cells. To initiate the differentiation of the cystoblast, BMP signaling must be decreased by different intrinsic and extrinsic mechanisms^8^. The majority of ECs limit the spreading of BMP ligands and therefore promote differentiation of the cystoblasts and dividing cysts^9,10^. Thus, the renewal somatic niche provides maintenance signals for GSCs, while a more posteriorly located differentiation niche, represented by ECs, is required for proper differentiation of GSC progeny.

The piRNA (Piwi-interacting RNA) pathway controls expression of transposable elements (TEs) in both somatic and germ cells of *Drosophila* ovaries. Piwi proteins guided by small piRNAs (24–30 nt) recognize complementary RNA molecules leading to their degradation or the repression of transcription with the help of other proteins (for review see^11^). The known molecular function of the piRNA pathway in the ovarian soma is the repression of a specific group of somatically active LTR retrotransposons^12–16^. The piRNA machinery in *Drosophila* ovarian somatic cells seems to be simpler than its counterpart in the germline. It operates via a single Piwi protein unlike the three proteins in germ cells and a substantial part of somatic piRNAs originates from a single source, the piRNA cluster *flamenco* (*flam*)^14,15,17^ that is an extended 180 kb region of X-chromosome heterochromatin, filled by TE copies and their fragments^18–20^. The *flam* locus is responsible for the repression of at least three somatically expressed retrotransposons: *gypsy*, *ZAM* and *Idefix*^21–24^. Cleavage of *flam* transcripts into small RNA molecules occurs in cytoplasmic Yb bodies. The cytoplasmic piRNA biogenesis machinery in somatic cells includes the nuclease Zucchini (Zuc), the RNA helicase Armitage (Armi), the TUDOR domain-containing proteins fs(1)Yb (Yb) and Vreteno (Vret), and other components^16,25–27^. In the course of *flam* transcript cleavage, piRNAs are loaded into Piwi and then move into the nucleus, where mature piRNA-Piwi complexes recognize complementary TE transcripts and repress their transcription with the help of adaptors, which recruit histone modification proteins, such as H3K9 methyltransferase Eggless (Egg) and H3K4 demethylase dLSD1^28–32^.

piRNA pathway mutations cause upregulation of TEs and lead to different oogenesis defects and sterility. Initially, two key components of the piRNA system, Piwi and Yb, have been shown to be required in somatic cells to prevent GSC loss^33,34^. Later it was found that the lack of several components of the somatic piRNA pathway, including Piwi^35–37^, Vret^27^, *flam*^23,38^ and Egg^38,39^ lead to the accumulation of undifferentiated germ cells in germaria, known as a germline tumor phenotype. The germ cell differentiation defects observed in piRNA pathway mutants are thought to be related to the dysfunction of ECs^36,37,39^. Knockdowns of Piwi and Yb specifically in ECs induced large numbers of ectopic GSC-like cells^36,37^. However, the underlying mechanisms are contradictory. Several papers noted an increased rate of somatic cell death in ovaries due to TE activation^27,38^. Others have found that Piwi downregulates expression of the *dpp* gene in ECs^36,37^ and that TE activation decreases the expression of Wnt4 ligand, which ensures EC function in germ cell differentiation^39^. It has been shown also that *piwi* mutations disrupt the spatial position of gonadal intermingled cells (the EC progenitors) and germ cells in early development^36^.

Here we provide results indicating that the germ cell differentiation defects caused by somatic TE activation in *flam* mutants are due to a decrease of EC precursor population at the larval stage, whereas no EC death or additional decline of their production rate was observed in *flam* adult ovaries. We also found drastic oogenesis defects in *flam* mutants combined with mutations of genes encoding Chk2 (Checkpoint kinase 2) or ATM (ataxia telangiectasia-mutated) checkpoint kinases, contrary to known suppressor effect of *chk2* mutation on ovarian development caused by TE derepression in the germline^38,40–43^. These results indicate that the somatic cells of ovaries are especially sensitive to TE upregulation upon loss of the Chk2 DNA damage response pathway.

## Results

### The occurrence of germ cell differentiation defects caused by somatic TE activation correlates with a reduced number of ECs

**Figure 1:**
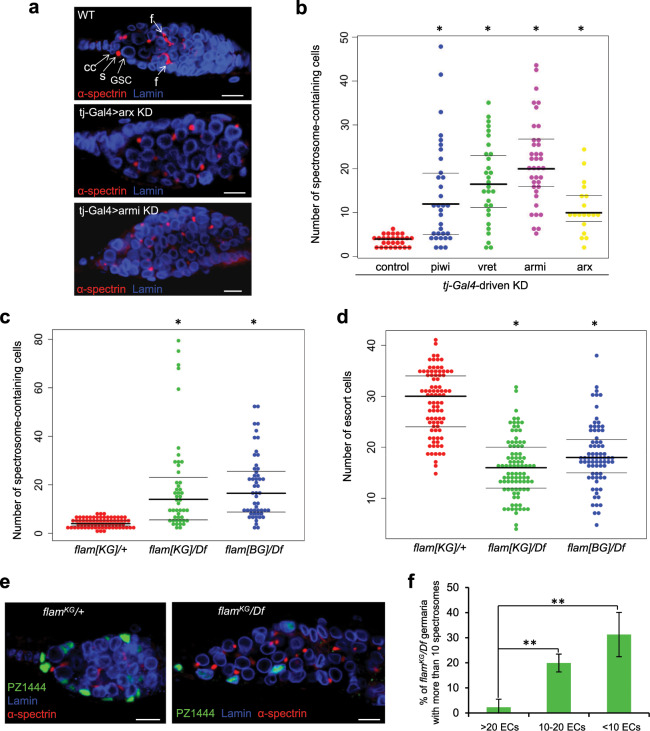
The occurrence of germ cell differentiation defects caused by somatic TE activation correlates with a reduced number of ECs. (**a**) Examples of wild-type and tumorous germaria stained for α-spectrin (red) to detect spectrosomes and fusomes and for lamin (blue) to visualize cell nuclei. A wild-type germarium (upper panel) usually contains 2–3 GSCs and a few cystoblasts marked by round spectrosomes (s). GSC are located at the anterior end of the germarium in close proximity to somatic cap cells (cc). Dividing cysts carry branched fusome structures (f). Germaria of Arx (middle panel) and Armi (lower panel) knockdowns (KDs) driven by *traffic jam Gal4* (*tj-Gal4*) in ovarian somatic cells carry an excess of spectrosome-containing cells and lack fusomes. (**b**) Quantification of spectrosome-containing cells in 7-day-old females with KDs of piRNA pathway components in ovarian somatic cells. Each dot corresponds to a single germarium. The central mark indicates the median, and the bottom and top lines indicate the 25th and 75th percentiles, respectively. All tested KD germaria contain significantly more spectrosomes than control (Mann–Whitney U-test; *p < 0.00001). Effects of Piwi and Vret KDs corroborate previously reported result^27,35–39^ (**c**). Quantifi**c**ation of spectrosome-containing cells in 7-day-old *flam* mutants (Mann–Whitney U-test; *p < 0.00001). (**d**) Quantification of ECs in *flam* germaria using *PZ1444-lacZ* line. EC number per germarium is indicated (Mann–Whitney U-test; *p < 0.00001). (**e**) Immunostaining of *flam*^*KG*^/*Df* and control germaria for α-spectrin (red), *PZ1444-lacZ* (green) and lamin (blue). (**f**) Increase of spectrosome-containing cell number in mutant germaria containing a reduced number of ECs. Percentage of *flam*^*KG*^/*Df* germaria with more than 10 spectrosome-containing cells in groups of germaria with different number of ECs is shown, based on three replicates (n = 181). Mean +/− s.d. are indicated. (Student’s t-test; **p < 0.05). Scale bars, 10 µm.

To extend previous observations^27,37–39^ that activation of TEs in ovarian somatic cells leads to germ cell differentiation defects, we estimated the spectrosome-containing cell number in ovaries lacking various components of the somatic piRNA pathway, some of which have not been tested in this regard before. For this and most subsequent experiments, we analyzed ovaries of 7-day-old females to allow tumor phenotype to develop to a pronounced degree. α-spectrin immunostaining revealed a drastic increase in the number of spectrosome-containing cells upon somatic depletion of Asterix (Arx) (also known as GTSF1) (Fig. 1a,b), a nuclear Piwi cofactor^29,30^. Depleting Armi, a cytoplasmic component of piRNA biogenesis machinery^16,25^, in all somatic cells of ovaries (Fig. 1a,b) or only in ECs (Fig. S1a) also caused germline tumors. Moreover, this phenotype was observed in ovaries lacking Zuc and Yb proteins (Fig. S1b). *piwi*^*Nt*^ mutation causing TE derepression due to cytoplasmic Piwi localization^44^ also led to the excess of spectrosome-containing germ cells (Fig. S1c), whereas in agreement with our previous report^44^ a GSC loss phenotype was rare in *piwi*^*Nt*^ ovaries in contrast to *piwi* null mutants (Fig. S1d). Thus, our results together with previous findings^27,38,39^, show that defects in germ cell differentiation are associated with the disruption of any component of the TE silencing pathway in somatic cells of ovaries.

Mutations affecting protein-coding genes could exert pleiotropic effects, if corresponding proteins have additional specific functions in oogenesis, unrelated to TE repression, as has been reported for Piwi^45,46^. Therefore, to directly examine the influence of activated somatic TEs on germline differentiation, we focused on the studies of *flam* piRNA cluster mutants. In most experiments, we analyzed *flam*^*BG*^*/Df* and *flam*^*KG*^*/Df* mutants carrying P-element-induced mutations^17,23^ and an X chromosome deletion (*Df*) covering the whole *flam* locus. Both mutants exhibit the derepression of *flam*-regulated somatic TEs (Fig. S2b) and about one-half of mutant germaria show a prominent germline tumor phenotype (Figs. 1c and S3a) and some other defects (Fig. S3b). We found no or faint *bam-GFP* reporter^4^ expression in germ cells constituting tumors in *flam*^*KG*^*/Df* germaria (Fig. S3c), indicating an abnormally enhanced BMP-signaling, which may be caused by the failure of ECs to restrict Dpp spreading^9,10^. The number of ECs visualized by immunostaining for *PZ1444 lacZ* reporter expression^47,48^ was reduced about two-fold from an average of 29 ECs per germarium in the *flam*/+ control to 16 and 18 ECs in *flam*^*KG*^*/Df* and *flam*^*BG*^*/Df* mutants, respectively (Figs. 1d and S3d). ECs of *flam* mutants lacked cellular processes that wrap up differentiating germ cells in wild-type ovaries (Fig. S3e). The latter effect may be a consequence of defective germline differentiation according to literature. For example, it has been shown that *bam* mutation impedes the formation of EC processes^9^.

Although the decrease in the EC number was previously reported for *piwi* somatic knockdown^37^, it was not clear whether EC reduction directly affects the germ cell differentiation. Since both the spectrosome and EC numbers substantially varied among individuals carrying *flam* mutations, we wondered how these parameters would be related within a single genotype. Simultaneous immunostaining of mutant ovaries with antibodies against α-spectrin and β-galactosidase (*PZ1444* reporter) (Fig. 1e) revealed that germline tumors were rarely detected in *flam* germaria containing more than 20 ECs, whereas germaria with a small number of ECs more often accumulated large numbers of spectrosome-containing cells (Fig. 1f). This result clearly shows a correlation between EC number reduction and germline tumor formation. Alternatively, the disruption of the differentiation niche may be caused by the loss of EC functional status, such as an abnormal or reduced production of signaling molecules. Impairment of different signaling pathways, including Wnt^35,49–51^, Rho^9,52^, EGFR^53,54^, Hh and Hpo/Yki^52^, as well as the enhancement of BMP signaling in ECs may lead to the germline tumor phenotype. Specifically, loss of piRNA pathway in ECs has been shown to be associated with enhancement of Dpp expression^36,37^ and a decrease in Wnt signaling^39^. However, we observed no significant changes in the Wnt2 ligand mRNA expression, a two-fold decrease of the Wnt4 ligand and Frizzled3 (Fz3, target of Wnt pathway) mRNAs and a slight upregulation of Dpp in both *flam*^*KG*^*/Df* and *flam*^*BG*^*/Df* germaria compared to control siblings (Fig. S4a). The two-fold decrease of *wnt4* expression is likely explained by the observed two-fold reduction of EC number in *flam* mutants (Fig. 1d), given that Wnt4 (but not Wnt2) is expressed only in ECs and is not detected in other cell types in the germarium^49^. The observed Dpp upregulation in *flam* germaria (Fig. S4a) can also be interpreted as a consequence of EC number reduction, because the antagonism between Wnt and BMP pathways in the ovarian somatic cells has been established^50,51^. The Wnt4 target *tkv-lacZ* expression^49^ was similar in ECs of *flam* and control ovaries indicating active Wnt signaling (Fig. S4b). Secreted Wnt ligands are known to act in ECs in an autocrine manner^49,51^ resulting in stabilization of a downstream effector protein β-catenin/Armadillo (Arm)^55^. We failed to find any alteration of Arm protein level in *flam* germaria by Western blot (Fig. S4c). Overexpression of Arm in *flam* mutants did not cause a decrease in spectrosome-containing germ cell number (Fig. S4d). Similarly, expression of the constitutive Arm form (*UAS-Arm-S10*) driven by *c587-Gal4* in ECs did not rescue the germline tumor phenotype of *piwi* mutants (Fig. S4e). As a whole, these results suggest that the germ cell differentiation defect in *flam* mutants is mediated rather by a decrease in the number of ECs, than by dysfunction of remaining ECs.

### EC number and the formation of germline tumor phenotype in flam mutants are determined at the larval stage

**Figure 2:**
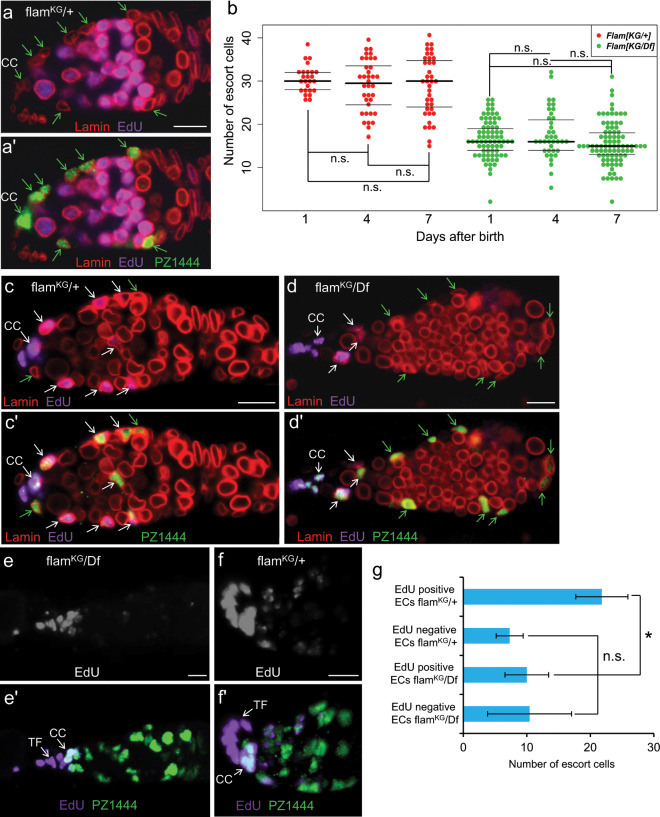
*flam* mutation leads to a reduction in EC number in larval development but not in adults. (**a**) *flam*^*KG*^/+ germarium of an adult female after feeding EdU for three days, stained for EdU (purple) and lamin (red). (**a’**) The same germarium with *PZ1444* immunostaining (green). ECs (indicated by green arrows) and CCs are EdU-negative. (**b**) Quantification of ECs in *flam*^*KG*^/+ (red dots) and *flam*^*KG*^/*Df* (green dots) flies at the age of 1, 4 and 7 days. The differences between samples of different ages of the same genotypes are not significant (n.s.) (Mann–Whitney U-test; p > 0.1). (**c,d**) Germaria of females, obtained from larvae reared on EdU-containing food. White and green arrows indicate EdU-positive and EdU-negative ECs, respectively. (**e**,**e’**) An example of *flam*^*KG*^/*Df* germarium with large number of ECs, most of which are EdU-negative after larval EdU incorporation. Full Z-series projections are shown. (**f**,**f’**) *flam*^*KG*^/+ germarium. EdU-positive ECs are located at the more anterior region of the germarium compared to EdU-negative ECs. (**g**) Quantification of EdU-positive and EdU-negative ECs in *flam*^*KG*^/+ and *flam*^*KG*^/*Df* germaria after larval EdU feeding. Mean +/− s.d. are indicated, based on three replicates (Student’s t test; *p = 0.01; n.s. = not significant). Scale bars, 10 µm.

ECs are initially produced from the intermingled cells during larval and pupal development^56–58^. Then, in adult ovaries ECs exhibit slow turnover rates, though a fraction of ECs is renewed. Escort stem cells^59^ or self-duplications of ECs^9,10^ were previously suggested as a source of new ECs in the adult gonads. A recent study revealed that new ECs in the imago are produced by divisions of follicle stem cells^60^. The reduction of EC number in *flam* germaria could be attributed to an increased rate of EC death, to defects of their renewal in adult ovaries or to a decline of EC production during earlier development. The TUNEL assay revealed less than 10% of *flam*/+ germaria containing at least one apoptotic EC. Unexpectedly, in *flam*^*KG*^*/Df* germaria the apoptotic ECs were even less frequently detected (Fig. S5a–c). To examine the formation of new ECs in *flam* ovaries, we carried out immunostaining for phosphohistone H3 Ser10 (PH3) mitotic marker and EdU incorporation assay. Both methods failed to detect a significant number of newly formed ECs in the *flam*^*KG*^*/Df* and control flies (Fig. S5d,e). Furthermore, most ovaries of females fed on EdU-containing food for three days did not contain EdU-positive ECs (Fig. 2a), suggesting that ECs in tested lines are mainly produced at earlier developmental stages. Importantly, we found about the same number of ECs in germaria of one-, four- or seven-day-old *flam* adults (Fig. 2b). Thus, the decrease of EC number is observed already in one-day-old *flam* mutants (Fig. 2b) and, therefore, is determined prior to the imago stage.

To find out the developmental stage when ECs are lost, we reared larvae on EdU-containing food, then placed eclosed flies on standard food and analyzed 3-day-old fly ovaries. In this case, all dividing larval cells will contain EdU signals, which then will be diluted with each round of replication in pupae and adults. Expectedly, the follicle cells and most of the germ cells were EdU-negative. Conversely, strong EdU immunostaining was observed in CCs and TF cells (Fig. 2c–f), which are known to be formed in larvae and then do not divide or renew^56–58,61^. About 70% of ECs were also labeled by EdU in *flam*^*KG*^/+ germaria. Apparently, the EdU-positive ECs were formed as a result of a few divisions of parental cells marked by EdU incorporation at the larval stage, while the EdU-negative ECs were likely produced later in development or originated from more actively proliferating cells. Interestingly, EdU-positive ECs were usually located more anteriorly than EdU-negative ECs (Fig. 2c,f), which is consistent with the possible origin of the latter from follicle stem cells^60^. In *flam*^*KG*^*/Df* germaria we observed a significant decrease of EdU-positive EC number compared to *flam*^*KG*^/+ sisters (Fig. 2d,e,g), which demonstrates decreased EC precursors formation in *flam* larvae. However, the number of EdU-negative ECs in *flam* mutant showed a large scatter of values (Fig. 2g). In some *flam* germaria the number of the EdU-negative ECs was even increased compared to control (as exemplified in Fig. 2e), suggesting that new ECs can be actively produced after the larval stage to compensate for the lack of EC precursors in earlier development.

**Figure 3:**
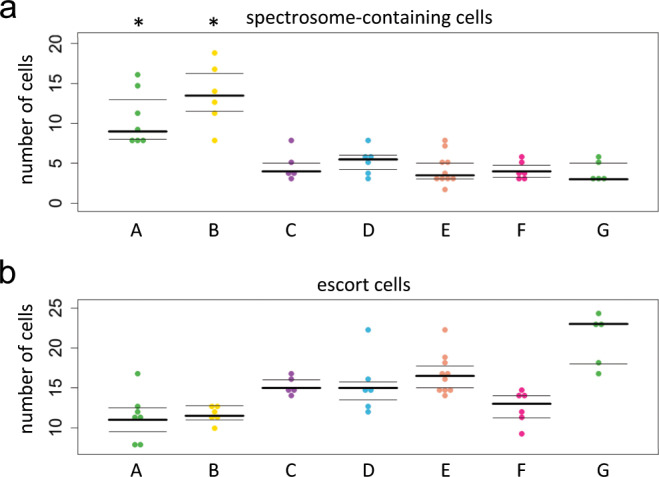
*flam* germaria belonging to the same ovary exhibit similar phenotypes. Quantification of spectrosome-containing cells (**a**) and ECs (**b**) per germarium in the same ovaries of *flam*^*KG*^/*Df* mutants. Germaria in one оvary are shown by grouped dots of the same color. Spectrosome numbers in germaria from tumorous ovaries (A and B) are significantly higher than their numbers in the non-tumorous ovaries (C-G) (Mann–Whitney U-test; *p < 0.05 for A and B vs C-G).

Primordial germ cells (PGCs) starting from mid-larval third instar stage are associated with intermingled cells that are EC progenitors. At this stage, all germ cells of the developing ovary are grouped together. Germaria formation occurs later in pupae, when TFs, CCs and their attached PGCs are separated into individual germaria units^56,62,63^. If EC number and germline differentiation defects are determined during larval development of *flam* gonads, a correlation can be expected between phenotypes of germaria within the same ovary. Indeed, we found that the numbers of both ECs and spectrosomes were quite similar in *flam* germaria belonging to the same ovary but varied substantially between individual ovaries (Fig. 3). Thus, developmental events prior to the pupal stage predetermine the germ cell differentiation defects in *flam* mutants.

### *flam* mutation induces DNA breaks in somatic cells of larval ovaries

**Figure 4:**
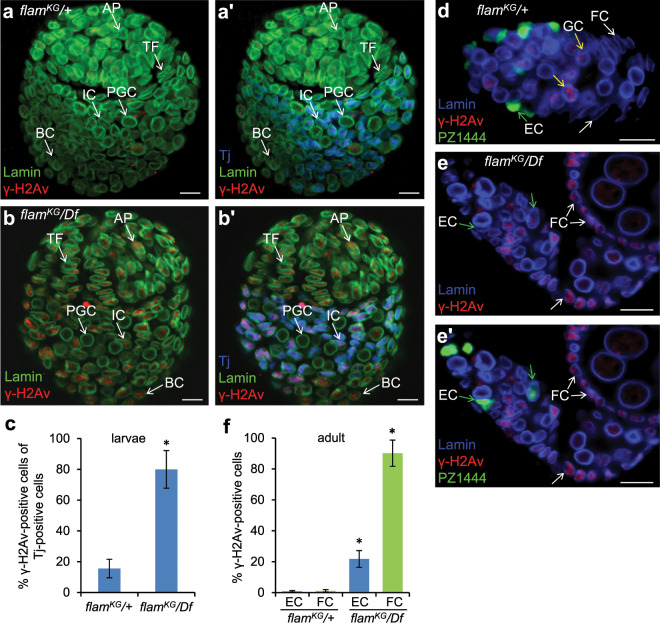
Intermingled cells in *flam* larval ovaries more often contain DNA breaks than ECs in adult ovaries. (**a**,**a’**) The *flam*^*KG*^/+ ovary of third instar larval stage stained for lamin (green), γ-H2Av DNA break marker (red) and Traffic jam (Tj, blue) showing intermingled cells (IC). Tj-negative cells include somatic apical (AP) and basal (BC) cells, TF, and Primordial germ cells (PGC). (**b**,**b**’) *flam*^*KG*^*/Df* L3 ovaries accumulate γ-H2Av in most ICs and other somatic cells, including TF and AP, but not in PGCs. (**c**) Quantification of γ-H2Av-positive among Tj-positive cells in *flam*^*KG*^/+ and *flam*^*KG*^/*Df* larval ovaries. Mean + /− s.d. are indicated (Student’s t test; *p < 1e-26). (**d**) *flam*^*KG*^/+ germarium of adult ovary stained for lamin (blue), γ-H2Av (red) and *PZ1444* EC marker (green). γ-H2Av signals are observed mainly in germ cells (GC, indicated by yellow arrows). (**e**,**e’**) In *flam*^*KG*^**/***Df* germaria γ-H2Av foci appear in most follicle cells (FC, white arrows) and only in some *PZ1444*-marked ECs (green arrows). (**f**) Quantification of γ-H2Av signals in FCs and ECs in ovaries of adults. Mean + /− s.d. are indicated (Student’s t test; *p < 1e-9). Scale bars, 10 µm.

The observed decline of EC precursor production in larval ovaries may be caused by the appearance of TE-induced DNA lesions in their genomes. To check this, we examined the presence of phosphorylated H2Av (γ-H2Av) histone, a commonly used DNA break marker^64^, in larval somatic intermingled cells marked by Traffic jam (Tj) immunostaining^56,58^. γ-H2Av dots were observed in 10–20% of Tj-positive cells in wild-type (*Batumi*) and *flam*^*KG*^/+ (Fig. 4a) third instar larval (L3) ovaries. In *flam*^*KG*^*/Df* L3 ovaries about 80% of Tj-positive cells contained γ-H2Av signals (Fig. 4b,c). γ-H2Av foci were also detected in Tj-negative somatic cells, including TF cells, as well as somatic apical (AP) and basal (BS) cells (Fig. 4b), which are known to be not incorporated into germaria^57^. However, most PGCs surrounded by intermingled cells did not contain γ-H2Av foci in mutant ovaries (Fig. 4b). Thus, the *flam* mutation leads to DNA breaks in somatic, but not germline cells of the larval ovaries. Immunostaining with activated Caspase3 antibodies, as well as TUNEL assay detected an increase of somatic cell death in *flam*^*KG*^*/Df* larval ovaries (Fig. S6). However, we cannot exclude that a reduction of EC number is partially caused by a decrease of division rate of EC precursors.

Then we monitored γ-H2Av presence in the somatic cells of adult *flam* ovaries. In *flam*/+ germaria, as in wild-type, γ-H2Av signals were absent in ECs and CCs, but were detected in the meiotic germ cells and endocycling nurse cells (Fig. 4d) where DNA breaks are generated during normal development^65–69^. In the *flam*^*KG*^*/Df* germaria only about 20% of ECs contained γ-H2Av foci, whereas follicle cells were mostly γ-H2Av-positive (Fig. 4e,f). These observations indicate that DNA damage events occur in mature *flam* ECs less often than in their precursors, intermingled cells, at the larval stage and/or mature ECs have an enhanced capacity to repair DNA lesions.

### The absence of Chk2 or ATM checkpoint kinases enhanced oogenesis defects of *flam* mutants

**Figure 5:**
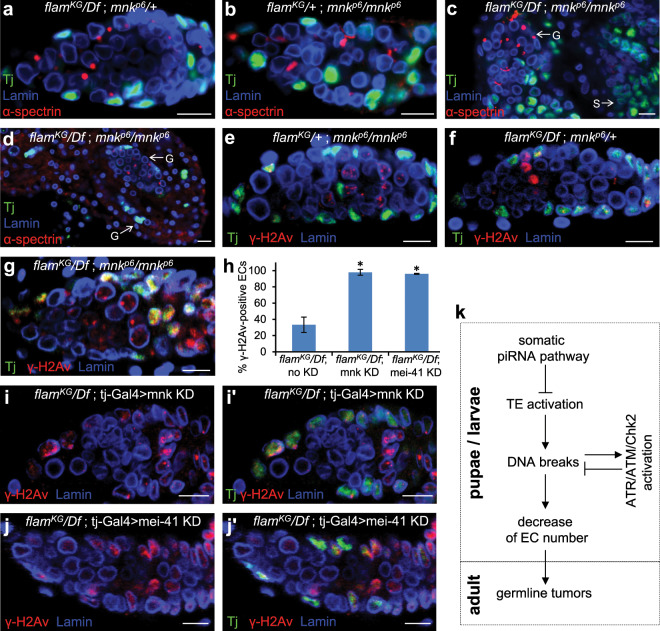
c*hk2* mutation enhances ovarian defects in *flam* mutants. (**a**) *flam*^*KG*^/*Df*; *mnk*^*p6*^/+ germarium stained for lamin (blue), α-spectrin (red) and Tj (green) showing nuclei of CCs, ECs and FCs. (**b**) *flam*^*KG*^/+*; mnk*^*p6*^*/mnk*^*p6*^ germarium with no morphological defects. (**c,d**) Fragments of *flam*^*KG*^/*Df*; *mnk*^*p6*^/*mnk*^*p6*^ ovaries showing an impaired formation of germaria and ovarioles. Abnormal germaria-like structures (G) are filled with spectrosome-containing cells and lack Tj-positive somatic cells or lack both germ cells and ECs. Separate accumulations of Tj-positive somatic cells (S) are indicated. (**e**) Immunostaining of *flam*^*KG*^/+; *mnk*^*p6*^/*mnk*^*p6*^ germarium with Tj (green), γ-H2Av (red) and lamin (blue). γ-H2Av signals are observed in meiotic germ cells and in a few somatic cells. (**f**) *flam*^*KG*^*/Df*; *mnk*^*p6*^/+ germarium containing γ-H2Av dots in follicle cells and in some ECs. (**g**) *flam*^*KG*^*/Df*; *mnk*^*p6*^/*mnk*^*p6*^ germarium showing increased intensity of γ-H2Av signals in Tj-positive somatic cells. (**h**) Quantification of γ-H2Av-positive ECs in *flam*^*KG*^*/Df*; *tj-Gal4/Cy* (no KD, control), *flam*^*KG*^*/Df*; *tj-Gal4* > *mnk KD* and *flam*^*KG*^*/Df*; *tj-Gal4* > *mei-41 KD* ovaries. Mean +/− s.d. are indicated (Student’s t test; *p < 0.001). **(i)** Immunostaining of *flam*^*KG*^*/Df*; *tj-Gal4* >*mnk KD* germarium with γ-H2Av (red) and lamin (blue) and Tj-marked somatic cells (green) (**i’)**. **(j,j’)** Immunostaining of *flam*^*KG*^*/Df*; *tj-Gal4* >*mei-41 KD* germarium with the same antibodies. (**k**) A working model of the occurrence of germ cell differentiation defects due to TE activation in ovarian somatic cells. Scale bars, 10 µm.

DNA damage is known to block cell proliferation through the activation of checkpoint kinases, which induce cell cycle arrest followed by apoptosis or DNA repair (for review see^70^). *Drosophila* Chk2 encoded by the *Mnk/Loki* gene together with other checkpoint kinases is required for cell cycle arrest in response to DNA breaks in both somatic and germ cells^71–74^. Another function of Chk2 is p53 phosphorylation that activates transcription of genes involved in DNA repair and/or apoptosis pathways^75,76^. To examine whether the *flam* mutant phenotype is mediated by the checkpoint response to TE-induced DNA breaks, we crossed the *mnk*^*p6*^ mutation (the well-characterized loss of function allele^42,43,71,77^) into a *flam* mutant background. Although the *chk2* mutation was shown to partially rescue the germline differentiation defects induced by TE activation in germ cells^38,42,43^, we unexpectedly observed its opposite effect in *flam* mutants. The *flam*^*KG*^*/Df*; *mnk*^*p6*^/*mnk*^*p6*^ double mutants had drastically more defective ovaries than *flam*^*KG*^*/Df*; *mnk*^*p6*^/+ individuals, whereas *flam*^*KG*^/+; *mnk*^*p6*^/*mnk*^*p6*^ ovaries displayed no visible morphological defects (Fig. 5a–d). The formation of germaria was abolished in most *flam*^*KG*^*/Df*; *mnk*^*p6*^/*mnk*^*p6*^ ovaries (Fig. 5c,d) and in some of them the number of Tj-positive ovarian somatic cells was highly reduced (Fig. 5d). Severe oogenesis defects were also observed when we combined *flam* with two different mutations in the *tefu* gene (Fig. S7), encoding a *Drosophila* homolog of ATM kinase that is directly recruited and activated by DNA double-strand breaks, acting upstream of Chk2^70,78^. We suggested that the observed catastrophic ovarian phenotypes can be induced by the death or dysfunction of ovarian somatic cells, including ECs due to their inability to repair TE-induced DNA lesions. Then, we checked whether DNA breaks are accumulated in ECs of *flam* mutants lacking checkpoint response. We found that somatic cell nuclei in rarely observed germaria-like structures of *flam*^*KG*^*/Df*; *mnk*^*p6*^/*mnk*^*p6*^ ovaries were dramatically enriched in γ-H2Av signals compared to both *flam*^*KG*^*/Df*; *mnk*^*p6*^/+ and *flam*^*KG*^/+; *mnk*^*p6*^/*mnk*^*p6*^ germaria (Fig. 5e–g). Somatic depletion of Mnk in the *flam*^*KG*^*/Df*, but not *flam*^*KG*^/+ background also caused accumulation of γ-H2Av in nearly all ECs (Fig. 5h,i) in contrast to about 20% of γ-H2Av-positive ECs observed in *flam*^*KG*^*/Df* ovaries (Fig. 4f). In addition, γ-H2Av foci were accumulated in ECs upon depletion of another checkpoint kinase, mei-41 (*Drosophila* homolog of ATR) (Fig. 5h,j), which, however, did not enhance oogenesis defects. Thus, Chk2, ATM and ATR kinases are involved in cellular response upon TE activation in ECs or their progenitors, but their specific molecular functions in this process warrant further examination.

## Discussion

Activation of TEs in ovarian somatic cells is known to compromise differentiation of germ cells^27,38,39^. Here, we found that the accumulation of GSC-like cells caused by mutations in the somatic piRNA cluster, *flam*, is determined by an insufficient number of somatic ECs. We demonstrated that the decrease of EC production in *flam* mutants, as well as the formation of germline tumor phenotype, depend on the events which occur in larvae and possibly at earlier stages of development, but not in the adult ovaries (Fig. 5k). These observations are consistent with previous report showing that Piwi expression in intermingled cells during the larval L3 stage is required to restrict GSC number in adults^36^. Of note, somatic piRNAs against TEs were found to be produced de novo in large amounts between embryogenesis and the L3 stage^79^ that shows the piRNA pathway activity in somatic cells of larval ovaries. Our finding of abundant DNA breaks in intermingled cells of *flam* mutant (Fig. 4b,c) indicates that activated TEs affect the genome of EC precursors at the larval stage. Interestingly, the intermingled cells accumulated more DNA breaks than mature ECs (Fig. 4). The reason for this vulnerability of somatic niche to TE activation during larval development remains unclear.

Mobilization of TEs in the germ cells was shown to initiate the checkpoint response. In developing oocytes, TE-induced DNA breaks trigger Chk2-dependent oocyte polarization abnormalities^40,41^. Consistent with this, *chk2* mutation suppresses polarization defects in the oocytes of piRNA pathway mutants^40,41^. Derepression of TEs in GSCs leads to Chk2-mediated arrest of cell cycle^42,43,80^ and induction of p53 activity^81^, which launches DNA repair or apoptosis. In particular, mutation of *aub* gene encoding a germline-specific piRNA-binding protein is phenotypically manifested as a decrease of GSC number and a delayed differentiation of cystoblasts^42,43^. The *chk2* mutation partially rescues these defects^42,43^. Transpositions of *P* element during hybrid dysgenesis also induce Chk2-dependent arrest of germ cell differentiation and selective apoptosis of some GSCs, whereas mutating Chk2 restores GSC self-renewal and normal looking germaria^38,80^. However, in this case *chk2* mutants show strong γ-H2Av signals and death of some cells at all oogenesis stages and never restore fertility^80^. Interestingly, GSCs in dysgenic females are able, over time, to acquire resistance to *P* element due to the piRNA amplification by ping-pong mechanism, whereas this adaptation does not occur in *chk2* mutants^80^. As a result, the ovarian defects in older dysgenic females are enhanced by *chk2* mutation^80^. Here, we for the first time examined the role of checkpoint response upon genomic stress caused by TE activation in somatic cells of ovaries, which lack ping-pong piRNA amplification^11,14,15^. We found that the absence of Chk2 or ATM kinases in the *flam* mutant background leads to dramatically more severe oogenesis defects compared to those induced by the *flam* mutation alone (Figs. 5 and S7). Thus, in contrast to germ cells, the Chk2-dependent response to TE activation in somatic ovarian cells is critical for the preservation of normal ovarian structure. The observed phenotypes of *flam mnk* double mutants indicate the loss of somatic cells due to the accumulation of unrepaired DNA lesions (Fig. 5). Our results suggest that the primary function of the Chk2-mediated response in ovarian somatic cells is the induction of DNA repair (Fig. 5k). The canonical activation of DNA repair/apoptosis pathways following DNA damage requires Chk2-mediated phosphorylation of p53^75,76^. However, p53 activity in *Drosophila* ovaries was shown to be restricted to GSCs and cystoblasts^81^, suggesting that in ovarian somatic cells Chk2 induces DNA repair by an unknown mechanism, which is of interest for further research.

## Methods

### Drosophila stocks

*Drosophila melanogaster* stocks were maintained under standard conditions at 25 °C. For analysis of *flam* mutations the following stocks were obtained from the Bloomington Drosophila Stock Center: *w*^1118^ *P{GT*1*}*^1^*, flam*^*BG02658*^ (#13912, *flam*^*BG*^), *y*^*1*^ *P{SUPor-P}flam*^*KG00476*^ (BDSC #16453, *flam*^*KG*^) and *Df(1)Exel6255*, *w*^1118^ *P{XP-U}Exel6255*/*FM7c* (BDSC #7723, *flamDf*). To distinguish between *flam*^*KG*^*/Df* and *flam*^*KG*^/+ larvae we used the *y*^1^ *w*^*67c23*^ *Alr*^1^/*FM7i, P{w[ + mC] = ActGFP}JMR3* (BDSC #25048) balancer and manually selected GFP-positive and GFP-negative larvae. To visualize ECs *flam* mutations were combined with the *PZ1444 lacZ* enhancer trap line^47,48^. For *piwi*, we used *piwi*^2^ and *piwi*^3^ null mutations^34^ and *piwi*^*Nt*^ mutation with disrupted Piwi nuclear localization^44^. To analyze *piwi*^*Nt*^*/piwi*^*Nt*^ females we used a strain with a higher survival rate of homozygous flies due to a change of genetic background. The following UAS-RNAi stocks were obtained from Vienna Drosophila Resource Center (VDRC): piwi-RNAi (#101658), vret-RNAi (#34897, #101134), armi-RNAi (#103589), arx-RNAi (#40480, #40479), mei-41-RNAi (#11251), Chk2-RNAi (#110342). RNAi depletion or expression of proteins was induced by *UAS-Dicer tj-Gal4* driver active in most somatic ovarian cells^16,82^ or *c587-Gal4* driver active in ECs and early follicle progenitors^9,37^. Other fly stocks were the following: *Df(2 L)Prl* and *zuc*^*HM27*^ (from T. Schüpbach lab), *mnk*^*p6*^ (*lok*^*p6*^)^71^ (from M. Simonelig lab), *tefu*^1^, *tefu*^*red3*1^ ^83^, *fs(*1*)Yb*^*1*^ (*Yb*^*1*^), *fs(1)Yb*^72^ (*Yb*^72^)^33^, *bamGFP*^4^, *Batumi*, *P{w(+mC) = UAS-arm.S10}C, y(1) w(1118)* (BDSC #4782, *UAS-arm.S10*), *y(1) w(1118); P{w(+mC) = UAS-arm.Exel}2* (BDSC #8369, *UAS-arm*), *y(1) w(67c23)*; *P{w(+mC) = lacW}tkv(k16713)*/*CyO* (BDSC #11191, *tkv-lacZ*), y(1) w(*); P{w(+mC) = UAS-mCD8::GFP.L}LL5 (BDSC #5137, *UAS-mCD8::GFP*).

### Immunostaining

For spectrosome analysis ovaries from 7-day-old females were used, and for other purposes - as indicated in the text. We revealed that germline differentiation defects in *flam* and *piwi* mutants are more pronounced in the progeny of older parents and to standardize further analysis we used offspring from the parents less than three weeks old. Immunostaining was basically performed as described previously^84^ with some modifications. Ovaries were manually isolated in PBT (PBS containing 0.01% Tween-20) at 4 °C, rinsed in PBS and fixed in 4% formaldehyde (in PBT) for 25 min at room temperature. Fixation was stopped by incubation with 0.25 M glycine (Sigma-Aldrich) for 5 min. Then ovaries were washed in PBS three times for 10 min at room temperature, permeabilized with PBTX (PBS with 0.1% Tween-20, 0.3% Triton X-100) for 10 min, blocked with PBTX containing 3% normal goat serum (NGS, Invitrogen) for 3 h, incubated with primary antibody in PBTX containing 3% NGS for 7 h at room temperature, or overnight at 4 °C, washed in PBTX three times for 10 min, incubated with secondary antibodies (1:1000) in PBTX containing 3% NGS for 7 h or overnight in a dark chamber, and then washed in PBTX three times for 10 min. Coverslips were mounted with a drop of SlowFade Gold Antifade reagent (Invitrogen) containing DAPI. The following primary antibodies were used: rabbit anti-lamin Dm0 (1:500, provided by P. Fisher^85^), chicken anti-β-galactosidase (1:500, Abcam, ab9361), mouse anti-β-galactosidase (1:200, DSHB #40-1а), rabbit anti-pS10H3 (1:200, Millipore #MC463), rabbit anti-GFP (1:500, Abcam, ab290), mouse anti-α-spectrin (1:200, DSHB, 3А9), rabbit anti-γ-H2av (1:100, Rockland, anti-H2AvD pS137), rat anti-Vasa (1:100; DSHB), guinea pig anti-Tj (1:5000, a gift from Dorothea Godt), rabbit anti-Caspase-3 antibody (1:200; Abcam, ab13847). The following secondary antibodies (Invitrogen, Thermo Fisher Scientific) were used: anti-rat IgG Alexa Fluor 546; anti-rabbit IgG Alexa Fluor 488; anti-rabbit IgG Alexa Fluor 546; anti-rabbit IgG Alexa Fluor 633; anti-mouse IgG Alexa Fluor 488; anti-mouse IgG Alexa Fluor 633; anti-chicken IgG Alexa Fluor 633; anti-guinea pig IgG Alexa Fluor 488; anti-guinea pig IgG Alexa Fluor 633. Confocal microscopy was done using LSM 510 META system (Zeiss).

### TUNEL assay

TUNEL staining was performed using Click-iT™ Plus TUNEL Assay for *In Situ* Apoptosis Detection, Alexa Fluor™ 647 dye kit (#C10619, Invitrigen, Thermo Fisher Scientific) according to the manufacturer’s instructions.

### EdU incorporation assays

For the two-hour EdU labeling, the ovaries were incubated in Grace’s medium containing 10 µM EdU for 2 hours at 25 °C. For the EdU *in vivo* incorporation assay, females were fed on food with yeast paste containing EdU (0.5 mM) for three days. For larval EdU assay, parental flies were placed on EdU-containing food (0.5 mM), where larvae developed. Then newly eclosed flies were placed on food without EdU and after 3 days the ovaries were dissected and analyzed.

The ovaries from all these types of assays were fixed, permeabilized as described above, and processed for EdU label detection using the Click-iT™ reaction according to the manufacturer’s instructions. Click-iT reaction was carried out in a cocktail containing Alexa Fluor 647 azide, triethylammonium salt (#A10277, Invitrogen) and Reaction Buffer Kit (#C10269, Invitrogen) 30 min in the dark at room temperature. Then ovaries were washed in PBTX and processed for immunostaining.

### Western blot

Ovarian lysates were fractionated by SDS-PAGE (10% acrylamide gel) and transferred to a PVDF membrane (Immobilon-P, Millipore). Blots were developed using alkaline phosphatase-conjugated secondary antibody (Sigma) and the Immun-Star AP detection system (Bio-Rad). The following primary antibodies were used: mouse anti-Arm (1:500, DSHB), and mouse anti-β-Actin (1:3000; Abcam, ab8224).

### RT-qPCR analysis

Total RNA was isolated from manually dissected ovaries using Trizol reagent (Invitrogen, Thermo Fisher Scientific) and cleared of genomic DNA by DNA-free kit (Ambion).

For analysis of signaling pathway genes in germaria, RNA was isolated from 0-1-day ovaries containing no late stage egg chambers. 1 μg of total RNA was used for the reverse transcription reaction with oligo(dT) primer and Superscript II reverse transcriptase (Invitrogen). The resulting cDNAs in at least three biological replicates were analyzed by RT-qPCR performed in MJ Mini thermal cycler (Bio-Rad) using SYBR Green chemistry (Applied Biosystems). The following primers were used for PCR:

Gypsy for CTTCACGTTCTGCGAGCGGTCT,

Gypsy rev CGCTCGAAGGTTACCAGGTAGGTTC,

Zam for3 TCACATCCTTCCAGCAATCTTCAA,

Zam rev3 TATTACAGTTTCTGACATTATTTCTTCGTG,

MDG1 dir AACAGAAACGCCAGCAACAGC,

MDG1 rev CGTTCCCATGTCCGTTGTGAT,

Idefix for AACAAAATCGTGGCAGGAAG,

Idefix rev TCCATTTTTCGCGTTTACTG,

dpp for2 GGCTTCTACTCCTCGCAGTG,

dpp rev2 TGCTTTTGCTAATGCTGTGC,

wnt4 for5 ATGATCCTCACCCACCTGAG,

wnt4 rev5 ACCTGACCAGCATTGTTTCC,

wnt2 for CAATAACCGAGCAGGGAGAAC,

wnt2 rev CATGAGTCTATCGCCAACCAG,

fz3 for TCTGCTTCGTCCTGACACTG,

fz3 rev CCTTGCTTGATTGTGGAACAC,

Rp49_up ATGACCATCCGCCCAGCATAC,

Rp49_rev2 GCTTAGCATATCGATCCGACTGG.

## Supplementary information

Supplementary information.

## Data Availability

All data generated or analyzed during this study are included in this published article (and its Supplementary Information Files).
