# Author Correction: Special vulnerability of somatic niche cells to transposable element activation in *Drosophila* larval ovaries

**DOI:** 10.1038/s41598-021-03387-5

**Published:** 2021-12-20

**Authors:** Olesya A. Sokolova, Elena A. Mikhaleva, Sergey L. Kharitonov, Yuri A. Abramov, Vladimir A. Gvozdev, Mikhail S. Klenov

**Affiliations:** 1 Department of Molecular Genetics of the Cell, Institute of Molecular Genetics, Russian Academy of Sciences, 2 Kurchatov Sq., 123182 Moscow, Russian Federation; 2 Present Address: Laboratory of Postgenomic Research, Engelhardt Institute of Molecular Biology, Russian Academy of Sciences, 32 Vavilova St., 119991 Moscow, Russian Federation

Correction to: *Scientific Reports* 10.1038/s41598-020-57901-2, published online 23 January 2020

The original version of this Article contained an error in the Acknowledgments section.

“The work was supported by the grant from Russian Foundation for Basic Research [16-04-01524 for M.K.] and by the Presidium of the Russian Academy of Sciences program Molecular and Cell Biology (for V. G.).”

now reads:

“This work was supported by the Russian Science Foundation (RSF) [grant number 19-14-00382 to M.S.K.].”

The original Article has been corrected.

