# Supplementary material for: Special vulnerability of somatic niche cells to transposable element activation in Drosophila larval ovaries
Source: Sci Rep. 2020 Jan 23;10:1076. doi: 10.1038/s41598-020-57901-2 (PMC6978372; doi:10.1038/s41598-020-57901-2)
Supplement: Supplementary file 1 — Supplementary information. [file 41598_2020_57901_MOESM1_ESM.pdf]

Supplementary Information

Special vulnerability of somatic niche cells to transposable element activation in *Drosophila* larval ovaries

Olesya A. Sokolova, Elena A. Mikhaleva, Sergey L. Kharitonov, Yuri A. Abramov, Vladimir A. Gvozdev, Mikhail S. Klenov

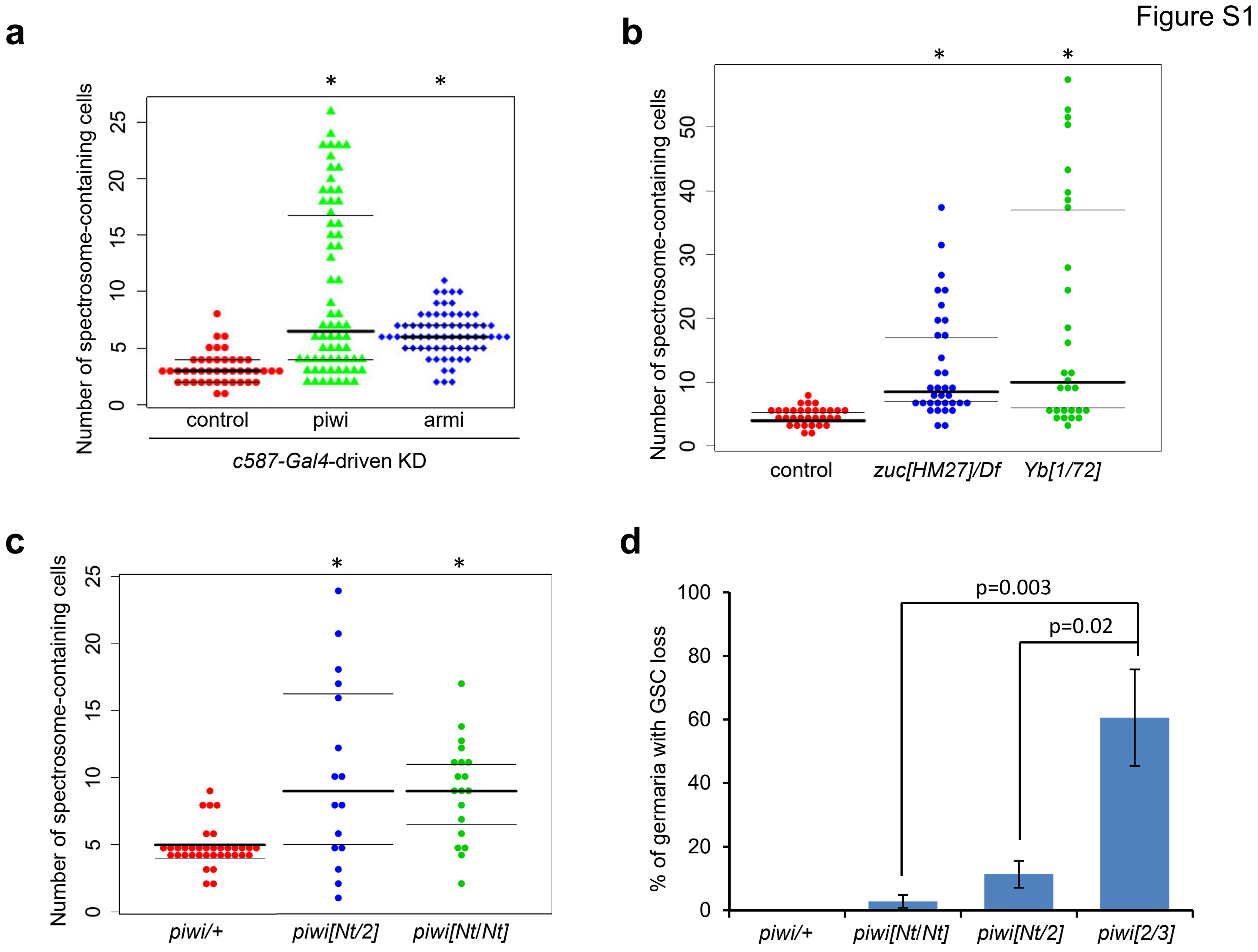

Figure S1. Disruption of somatic piRNA pathway induces germline tumor phenotype. Quantification of spectrosome-containing cells in germaria: (**a**) *piwi* and *armi* knockdowns driven by *c587-Gal4* in ECs; (**b**) *zuc* and *fs(1)Yb* mutants; (**c**) $piwi^{Nt}$ ovaries carrying cytoplasmic Piwi. Empty germaria without spectrosomes (zero values) that are often found in *fs(1)Yb* mutants are not included in the graphs. The central mark indicates the median, and the bottom and top lines indicate the 25th and 75th percentiles, respectively. Mutant germaria contain significantly more spectrosomes than corresponding controls (Mann–Whitney U-test; $^*p < 0.00001$). (**d**) Average percentage of germaria with no spectrosome-containing cells (GSC loss phenotype) is shown for $piwi^{Nt}/piwi^{Nt}$ (n=35), $piwi^{Nt}/piwi^{2}$ (n=41) and $piwi^{2}/piwi^{3}$ (null mutants) (n=64). This phenotype was not observed in *piwi*/+ control (n=52). Mean +/- s.d. and p-values from Student's t-test are indicated, based on three replicates.

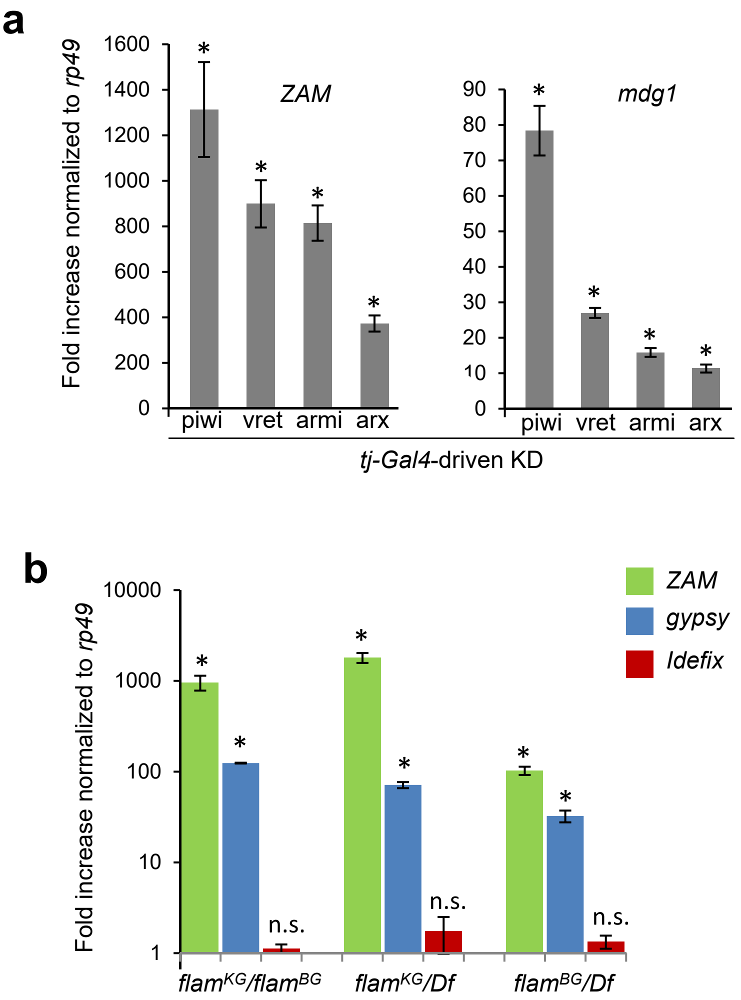

Figure S2. RT-qPCR of TE expression in ovaries of knockdowns and mutants of the studied piRNA pathway components. (**a**) Fold increase of *ZAM* and *mdg1* transcript levels in *tj-Gal4* mediated KDs of Piwi, Vret, Armi and Arx, normalized to control ovaries and *rp49* mRNA. Mean +/- s.d. are indicated (Student's t-test; *p < 0.00001). (**b**) Fold increase of *ZAM*, *gypsy* and *Idefix* transcript levels in *flam* mutant ovaries relative to heterozygous sisters and *rp49* mRNA. Mean +/- s.d. are indicated (Student's t-test; *p < 0.00001; n.s. = not significant).

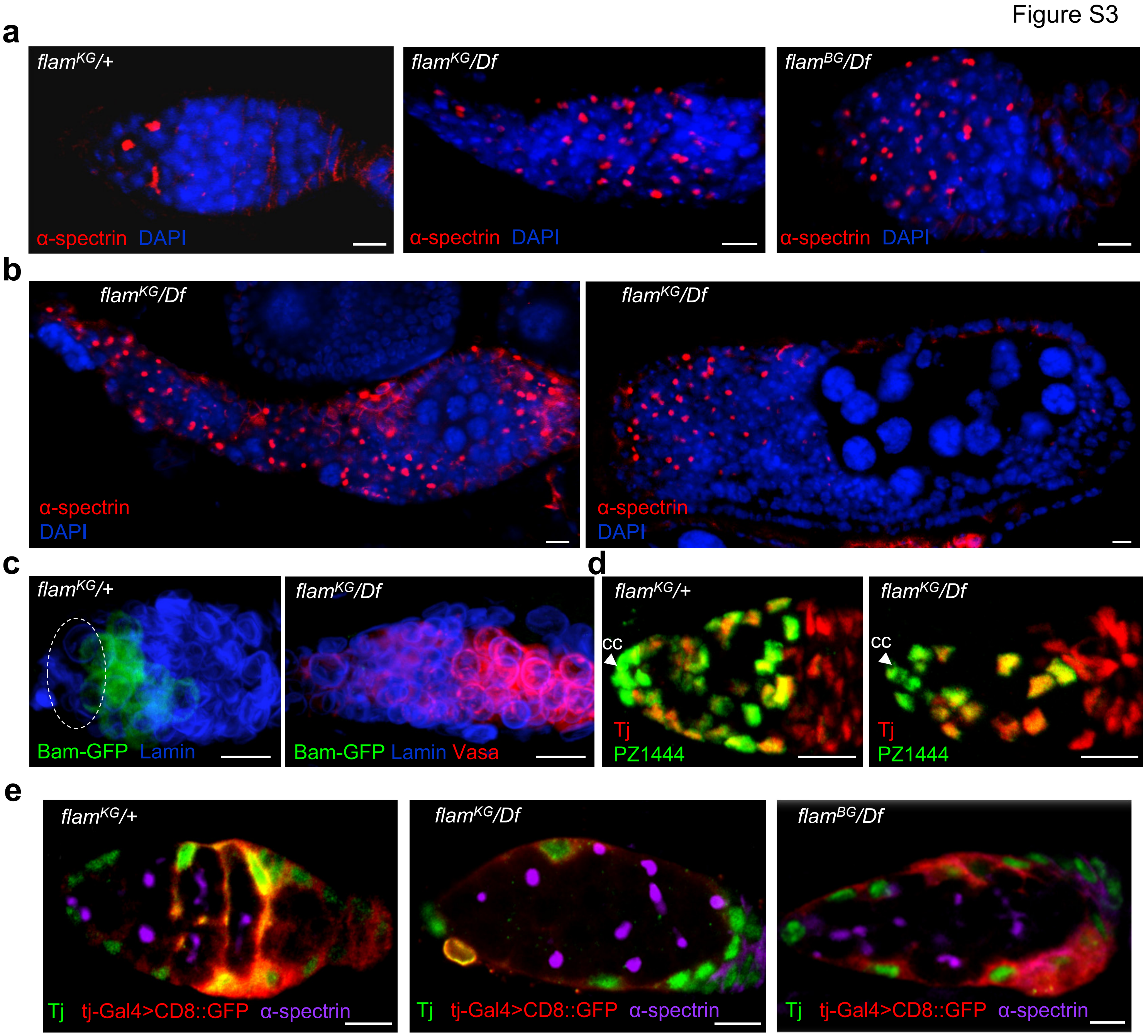

Figure S3. Accumulation of GSC-like cells in ovaries of *flam* mutants. (**a**) Immunostaining of the control *flam*/+ and tumorous *flam*^{KG}/*Df* and *flam*^{BG}/*Df* mutant germaria with DAPI (blue) and anti-α-spectrin (red) showing spectrosomes and fusomes. (**b**) Examples of abnormal and fused *flam* egg chambers with the expansion of spectrosome-containing cells. Immunostaining for α-spectrin (red). (**c**) Left panel: control germaria, the *Bam-GFP* reporter (green) is repressed in GSCs (indicated by a dotted circle) and is upregulated in CBs and mitotic cysts. Right panel: the *flam*^{KG}/*Df* mutant, germ cells detected by Vasa staining (red) show no expression of *Bam-GFP*. (**d**) Full Z-series projections of *flam*^{KG}/*Df* and *flam*^{KG}/+ germaria stained for Tj (red) to visualize all ovarian somatic cells and for β-galactosidase (green) to detect *PZ1444* expression in CCs and ECs. Most β-gal-positive cells are ECs with the exception of several cap cells (CCs) at the anterior end of the germarium (white arrowheads). (**e**) ECs of *flam* mutants exhibit defects of cellular processes that wrap germline cysts. Immunostaining for GFP to visualize membrane-tethered *UAS-CD8-GFP* reporter (red) in EC cellular processes; for Tj (green) to mark nuclei of ovarian somatic cells and for α-spectrin (purple).
Scale bars, 10 μm.

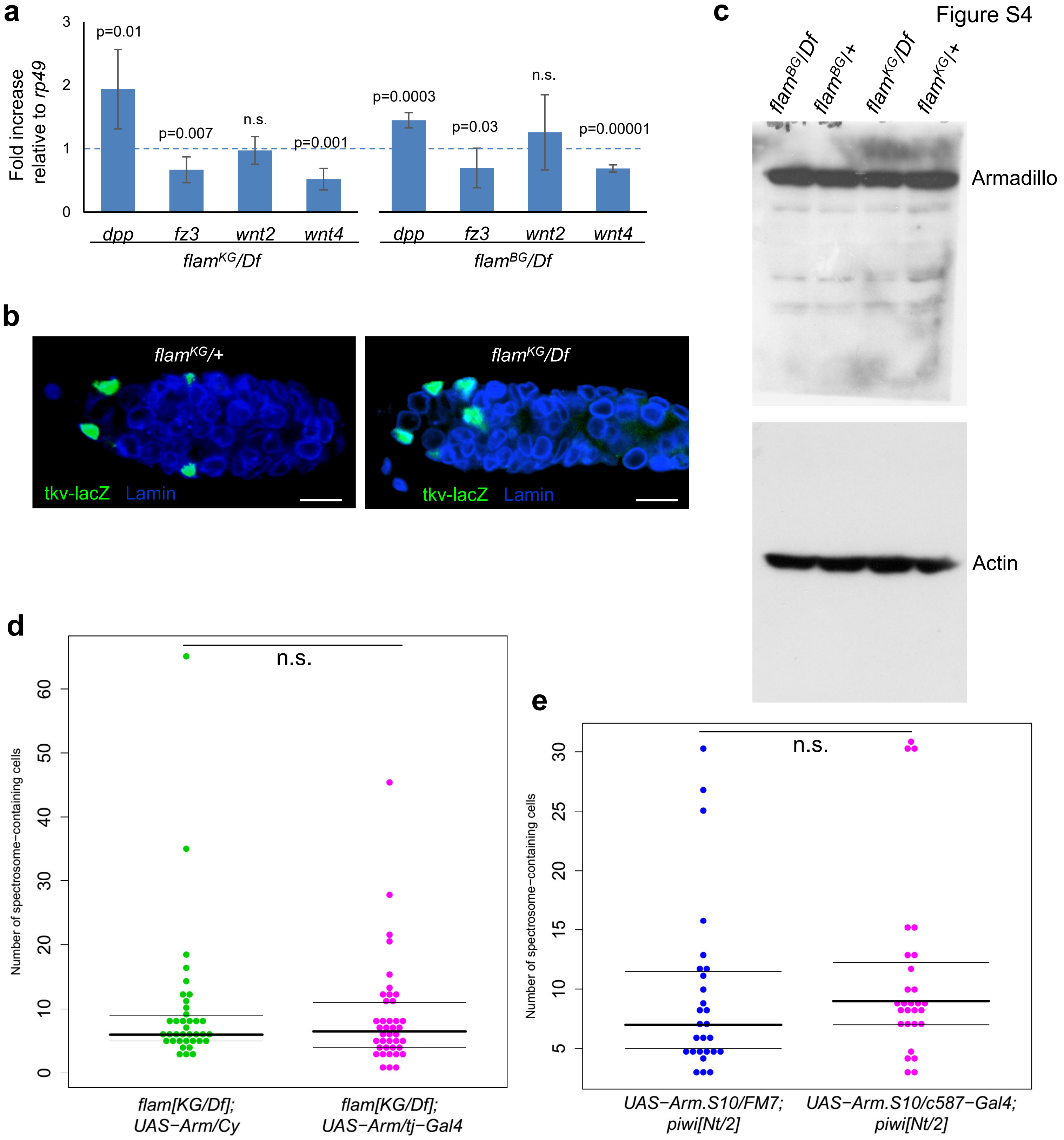

Figure S4. Analysis of relationship between Wnt signaling and the germ cell differentiation defects in *flam* mutants. (**a**) RT-qPCR quantification of *dpp* transcripts and components of Wnt pathway in 0-1-day-old ovaries of *flam* mutants. RNA was isolated from ovaries containing no late stage egg chambers, which may interfere with the detection of differences in the expression of these RNAs in the germaria. Bars show fold change of transcript abundance relative to control siblings obtained in the same crosses, normalized to *rp49* mRNA (n=5 for $flam^{KG}/Df$ and $flam^{KG}/+$ siblings; n=3 for $flam^{BG}/Df$ and $flam^{BG}/+$). Mean +/- s.d. and p-values from Student's t-test are indicated. n.s. = not significant. (**b**) The Wnt4-activated reporter *tkv-lacZ* expression in ECs of the *flam* mutant and control ovaries. Immunostaining for β-galactosidase (green) and lamin (blue). ECs are detected by their location and nuclei shape. Scale bars, 10 µm. (**c**) Western blot for Armadillo (Arm) protein in $flam^{BG}/Df$, $flam^{BG}/+$ and $flam^{KG}/Df$ and $flam^{KG}/+$ ovaries. Actin is used as a loading control. Full-length blots are shown. (**d, e**) Quantification of spectrosome-containing cells in germaria with Arm overexpression in *flam* mutants (**d**) and expression of the constitutive Arm form (*UAS-Arm-S10*) driven by *c587-Gal4* in ECs of $piwi^{Nt}/piwi^{2}$ mutants (**e**). Differences between germaria with Arm overexpression (purple dots) and mutant siblings without *tj-Gal4* or *c587-Gal4* drivers (green and blue dots, respectively) are not significant (n.s.) (Mann–Whitney U-test; p > 0.1).

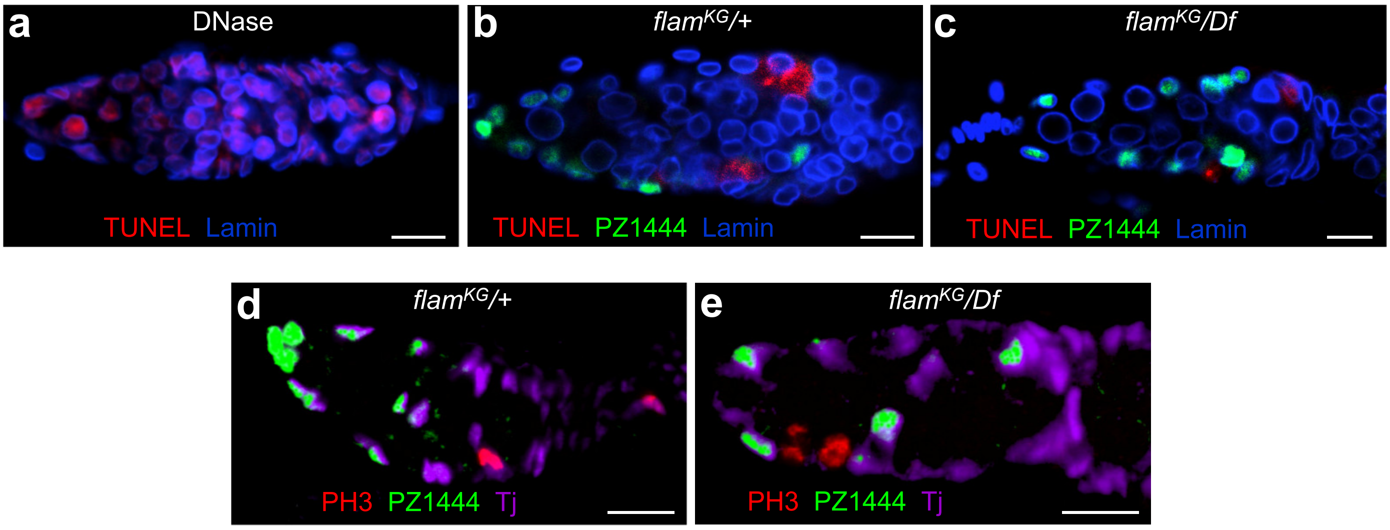

Figure S5. Escort cells are quiescent in adult ovaries of the *flam* mutant. (**a-c**) *flam*/+ and *flam^{KG}/Df* germaria stained for TUNEL (red), PZ1444 (green) and lamin (blue). (**a**) DNase treatment control showing TUNEL signals in the nuclei of all cells. **(b, c)** *flam*/+ and *flam^{KG}/Df* germaria normally contain some TUNEL-positive somatic and germ cells, but not ECs. Apoptotic ECs were observed in three *flam*/+ germaria (n=54) and none in *flam^{KG}/Df* germaria (n=67). (**d, e**) *flam*/+ and *flam^{KG}/Df* germaria stained for PH3 (phospho-histone H3 Ser10, red), PZ1444 (green) and the somatic cell marker Tj (purple). PH3 signals are observed in dividing cysts and in a fraction of follicle cells, but not in ECs (n=81 for *flam*/+ and n=72 for *flam^{KG}/Df* germaria).
Scale bars, 10 µm.

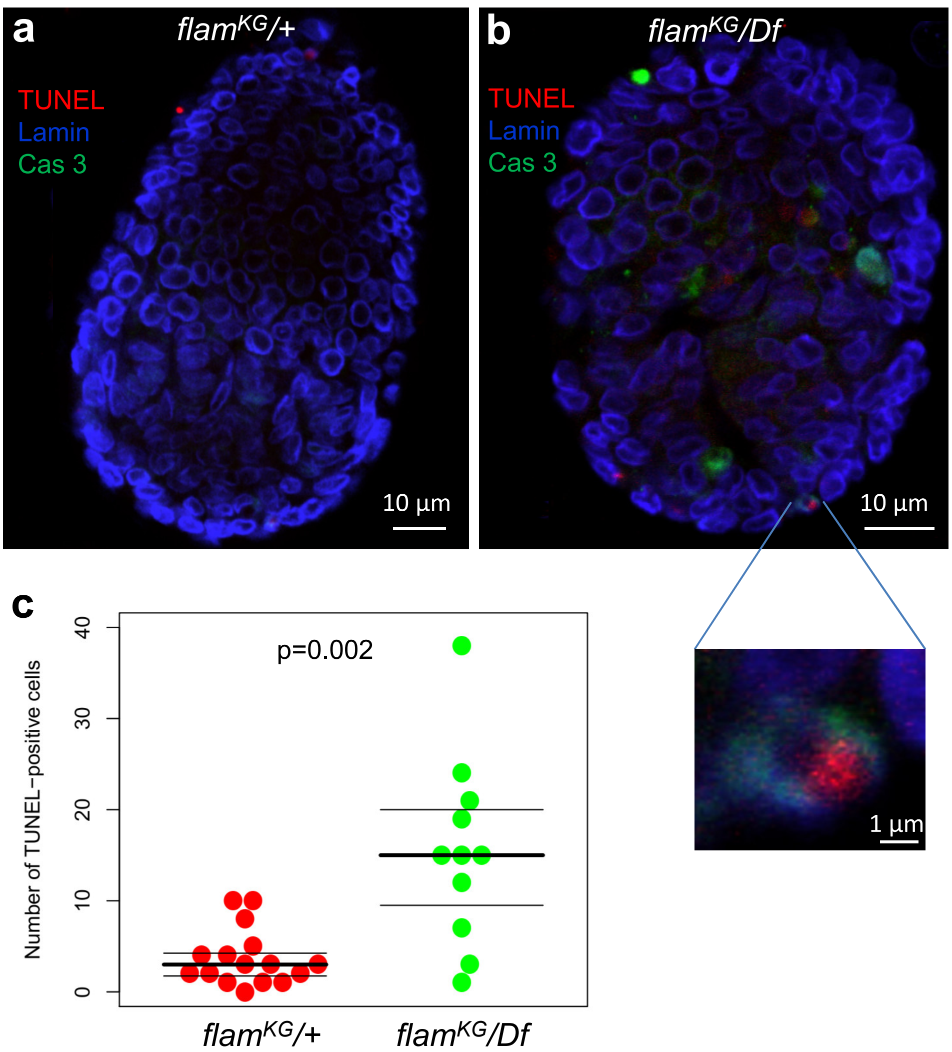

Figure S6. A rate of somatic cell death is increased in larval ovaries of *flam* mutants. (**a, b**) *flam*/+ and *flam^{KG}/Df* larval ovaries stained for TUNEL (red), activated caspase 3 (green) and lamin (blue). An insert shows an apoptotic cell with the TUNEL-positive nucleus and caspase 3 in the cytoplasm. (**c**) The number of somatic cells of all types with TUNEL signal per larval L3 ovary. The central mark indicates the median, and the bottom and top lines indicate the 25th and 75th percentiles, respectively. The difference between *flam*/+ and *flam^{KG}/Df* larval ovaries is significant (Mann–Whitney U-test; p = 0.002).

Figure S7

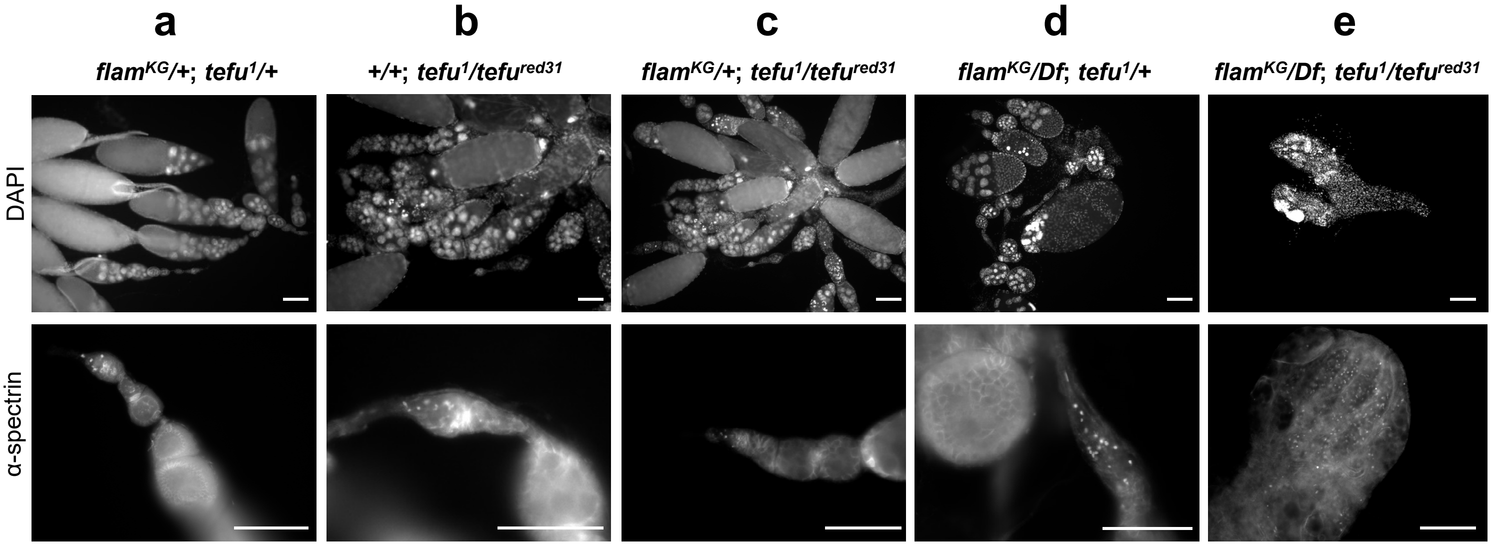

Figure S7. Mutations of ATM kinase induce drastic ovarian defects in *flam* mutant background. Upper panel: ovarioles of *tefu* mutants, *tefu*; *flam* double mutants and their heterozygous siblings stained with DAPI. Lower panel: ovarioles stained for α-spectrin. (**a**) Ovarioles of *flam*^{KG}/+; *tefu*^{1}/+ heterozygotes showing no visible morphological defects. (**b**) *tefu*^{1}/*tefu*^{red31} and (**c**) *flam*^{KG}/+; *tefu*^{1}/*tefu*^{red31} ovarioles showing mild defects, including some elongated egg chambers and eggs without appendages. (**d**) *flam*^{KG}/*Df*; *tefu*^{1}/+ displaying the characteristic *flam* phenotype. (**e**) *flam*^{KG}/*Df*; *tefu*^{1}/*tefu*^{red31} ovaries containing few enlarged germaria-like structures filled with large numbers of spectrosome-containing cells and lacking egg chambers.
Scale bars, 100 μm.
